# Skin cancer in essential thrombocythaemia and polycythaemia vera patients treated with hydroxycarbamide

**DOI:** 10.1002/jha2.551

**Published:** 2022-09-02

**Authors:** Abhinav Mathur, Joakim Edman, Livia Liang, Neil W. Scott, Henry G. Watson

**Affiliations:** ^1^ Department Haematology Aberdeen Royal Infirmary Foresterhill Health Campus Aberdeen UK; ^2^ School of Medicine Medical Sciences and Nutrition Aberdeen University Aberdeen UK; ^3^ National University Health System National University Hospital Singapore Singapore; ^4^ Institute of Applied Health Sciences University of Aberdeen, Polwarth Building, Foresterhill Aberdeen UK

**Keywords:** essential thrombocythaemia, hydroxycarbamide, melanoma, myeloproliferative neoplasm, non‐melanoma skin cancer, polycythaemia vera

## Abstract

Hydroxycarbamide (HC) is used as a cytoreductive treatment in myeloproliferative neoplasms (MPN). Observational studies have raised the possibility that HC contributes to the development of secondary malignancies, including skin tumours in MPN patients. In this retrospective observational study, we report a single‐centre experience of 324 HC‐treated MPN patients with long‐term follow‐up, compared to 47 MPN patients not on HC. Thirty‐three patients (10.2%) (HC) versus one patient (2.1%) (no HC) developed skin tumours during follow‐up (Hazard ratios [HR] 5.70, 95% confidence intervals 0.66–48.09, *p* = 0.112). However, male gender, age at MPN diagnosis, type of MPN (polycythaemia rubra vera) and previous history of skin cancer were prognostic variables associated with development of skin cancer.

## 1 INTRODUCTION

Hydroxycarbamide (HC) is the first‐line cytoreductive agent used to manage high‐risk essential thrombocythaemia (ET) [1] and polycythaemia rubra vera (PRV) [2]. HC is a non‐alkylating antimetabolite that interferes with cellular DNA synthesis and repair by inhibiting ribonucleoside diphosphate‐reductase [3].

HC is well‐tolerated with most of its side effects being uncommon, mild and reversible. However, observational studies conducted have suggested a higher incidence of secondary malignancies in patients with myeloproliferative neoplasms (MPN) such as ET and PRV [4, 5, 6]. Notably, an increased incidence of non‐melanoma skin cancers (NMSC) in HC‐treated patients has been reported, particularly in older men [7, 8]. However, one large study of ET patients with a 30‐year follow‐up period did not find any association between HC exposure and development of secondary malignancy in the group as a whole. Male gender and age >60 years were the only factors correlated with higher risk [9].

It is unclear whether the increased incidence of secondary cancers is due to the underlying MPN or to the therapies used to treat them. In this retrospective study, we report incidence of skin cancer and contributory risk factors for its development in a large cohort of ET and PRV patients from a single‐centre treated with HC with long‐term follow‐up.

### 1.1 Patients and methods

The Haematology Department at Aberdeen Royal Infirmary has maintained a database of MPN patients with the earliest records dating from 1976. These patients are routinely reviewed in specialist clinics. The frequency of review varied with some patients requiring more frequent follow‐ups, but no patient included in the study was seen less than once a year. To be included, patients needed a diagnosis of ET or PRV according to standard diagnostic criteria.

Patients were retrospectively identified by reviewing paper and electronic (InterSystems TrakCare Patient Management System) records. Data collected included basic demographics, dates of MPN and skin cancer diagnoses, driver mutations, duration of HC therapy (time from which patient started HC until stopped or censor date reached) and date of death. The definition of HC use included any HC use, either immediately following diagnosis or at any point in subsequent follow‐up.

Records for all patients were available for review at the end of the follow‐up period. The primary research question was into the effect of HC use on skin cancer. All‐cause mortality was assessed as a secondary research question. Patients were censored at the date of last follow‐up (30/05/22) or on the date of death (skin cancer study only).

Ethical approval for this project was granted from the NHS Grampian Caldicott Guardian (CG/2020/59).

### 1.2 Statistical analysis

All statistical analyses were performed using IBM SPSS Statistic package (version 27). Time to skin cancer and death were described using Kaplan–Meier analysis. Two separate Cox regression models were performed for skin cancer and all‐cause mortality. Hazard ratios (HR) and 95% confidence intervals (95% CIs) were estimated for HC use using Cox regression modelling adjusted for gender, age, type of MPN at diagnosis, JAK2 mutation status and previous history of skin cancer. To ensure that the assumptions of proportional hazards were met, log‐minus‐log plots were constructed. Statistical significance was set at the 5% level.

## 2 RESULTS

### 2.1 Patient cohort

A total of 371 patients who had a diagnosis of PRV (*n* = 138) or ET (*n* = 233) were identified. The majority of patients were female (56.1%) and had an underlying JAK2 V617F mutation (71.9%). The median age at MPN diagnosis was 66.0 years (interquartile range [IQR] 56–66). Seventeen patients experienced transformation of their MPN (seven to myelofibrosis, seven to PRV, one to acute lymphoblastic leukaemia, one to acute myeloid leukaemia and one to MPN/MDS crossover). No patients were lost to follow‐up. Median follow‐up for the cohort was 7.2 years (IQR 4–12). One hundred nineteen patients had follow‐up for ≥10 years.

Three hundred twenty‐four patients (87.3%) were prescribed cytoreductive HC, while 47 (12.6%) were not. The differences between these treatment groups are summarised in Table 1. There were proportionally more PRV patients in the non‐HC group. The median duration of HC treatment was 5.4 years (IQR 3–10).

**TABLE 1:** Clinical characteristics of the cohort

Variable	Total patients	Hydroxycarbamide	Non‐hydroxycarbamide
**Number of patients**	371	324 (87.3)	47 (12.6)
**Gender, *n* (%)**			
Male	163 (43.9)	138 (42.6)	25 (53.2)
Female	208 (56.1)	186 (57.4)	22 (46.8)
**Median age, years (range)**	66 (19–93)	67 (19–93)	57 (22–84)
**MPN, *n* (%)**			
PRV	138 (37.2)	104 (32.1)	34 (72.3)
ET	233 (62.8)	220 (67.9)	13 (27.7)
**Mutation status, *n* (%)**			
None identified	57 (15.4)	48 (14.8)	9 (19.1)
JAK2 V617F	265 (71.4)	233 (71.9)	32 (68.1)
JAK2 Exon 12	2 (0.5)	–	2 (4.3)
CALR	38 (10.2)	34 (10.5)	4 (8.5)
MPL	6 (1.6)	6 (1.9)	–
Multiple mutations	3 (0.8)	3 (0.9)	–
**Transformation, *n* (%)**			
Overall	**17 (4.6)**	**14 (4.3)**	**3 (6.4)**
Myelofibrosis	7 (1.9)	6 (1.9)	1 (2.1)
PRV	7 (1.9)	5 (1.5)	2 (4.3)
AML	1 (0.3)	1 (0.3)	–
ALL	1 (0.3)	1 (0.3)	–
MPN/MDS crossover	1 (0.3)	1 (0.3)	
**Skin cancer, *n* (%)**			
Overall	**34 (9.2)**	**33 (10.2)**	**1 (2.1)**
Melanoma	3 (0.8)	3 (0.9)	–
Basal cell carcinoma	15 (4.0)	14 (4.3)	1 (2.1)
Squamous cell carcinoma	4 (1.1)	4 (1.2)	–
Bowen's disease	4 (1.1)	4 (1.2)	–
Multiple skin cancers	8 (2.2)	8 (2.5)	–

Abbreviations: ALL, acute lymphoblastic leukaemia; AML, acute myeloid leukaemia; ET, essential thrombocythaemia; MDS, myelodysplastic syndrome; MPN, myeloproliferative neoplasm; PRV, polycythaemia rubra vera.

### 2.2 Skin cancer by HC use

Thirty‐four patients (9.2%) developed skin cancer during follow‐up: 15 had basal cell carcinoma (BCC), four had squamous cell carcinoma (SCC), four had Bowen's disease, three had melanoma, and eight had multiple skin tumours (combinations of BCC, SCC and Bowen's). Sixteen patients had a history of skin cancer before commencing HC therapy. Only one case (2.1%) of skin cancer was observed in the non‐HC treated group. Out of 324 patients that had received HC, 33 (10.2%) developed skin cancer.

The median age at first skin cancer diagnosis was 80.5 years (IQR 72–85). The mean skin cancer‐free survival was 37.7 years (95% CI 33.4 – 42.0 years). There was no evidence that HC use was associated with skin cancer (HR 5.70 95% CI 0.66–48.90, *p* = 0.112). However, age ≥60 years at MPN diagnosis (≥60 years vs. < 60 years, HR 9.70, 95% CI 2.81–33.47, *p* = 0.001), male gender (HR 2.21, 95% CI 1.05–4.63, *p* = 0.019), PRV (ET vs. PRV, HR 0.35, 95% CI 0.13–0.91, *p* = 0.032) and previous history of skin cancer (HR 3.86 95% CI 1.53–9.73, *p* = 0.004) were associated with the development of skin cancer (Table 2).

**TABLE 2:** Cox regression analysis of time to first skin cancer

Variable	Hazard ratio + 95% confidence interval	*p‐*Value
**Use of hydroxycarbamide**		
No	1	0.112
Yes	5.70 (0.66–48.90)	
**Age at MPN diagnosis**		
<60 years	1	**0.001**
≥60 years	9.70 (2.81–33.47)	
**MPN**		
PRV	1	**0.032**
ET	0.35 (0.13–0.91)	
**Mutation status**		
Mutation negative	1	0.160
JAK2 mutations	0.36 (0.12–1.08)	0.068
Other mutations	0.84 (0.27–2.58)	0.761
**Gender**		
Female	1	**0.036**
Male	2.21 (1.05–4.63)	
**Previous history of skin cancer**		
No	1	**0.004**
Yes	3.86 (1.53–9.73)	

Abbreviations: ET, essential thrombocythaemia; MPN, myeloproliferative neoplasm; PRV, polycythaemia rubra vera.

Death and reaching censor date are treated as censoring events for time to first skin cancer analysis.

### 2.3 All‐cause mortality

There were 33 deaths during the follow‐up period. The median survival of the whole cohort was 36.7 years (95% CI 33.0–41.5, supplementary data). There was no evidence that HC use was associated with increased mortality (HR 1.15, 95% CI 0.30–4.32, *p* = 0.840). Age at MPN diagnosis was the only significant variable affecting mortality (HR 10.2, 95% CI 2.90–35.85, *p* = 0.001) (Table 3).

**TABLE 3:** Cox regression analysis of time to mortality

Variable	Hazard ratio + 95% confidence interval	*p‐*Value
**Gender**		
Female	1	0.223
Male	1.57 (0.76–3.23)	
**Age at MPN diagnosis**		
<60 years	1	**0.001**
≥60 years	10.19 (2.90–35.85)	
**MPN**		
PRV	1	0.717
ET	0.86 (0.39–1.93)	
**Mutation status**		
Mutation negative	1	0.862
JAK2 mutations	1.01 (0.38–2.68)	0.979
Other mutations	0.71 (0.17–2.95)	0.638
**Use of hydroxycarbamide**		
No	1	0.840
Yes	1.15 (0.30–4.32)	
**Previous history of skin cancer**		
No	1	0.679
Yes	0.65 (0.09–4.96)	

Abbreviations: ET, essential thrombocythaemia; MPN, myeloproliferative neoplasm; PRV, polycythaemia rubra vera.

Reaching censor date was only censoring event for time to mortality analysis.

## 3 DISCUSSION

Skin cancer is the most significant cutaneous side effect reported with HC use. British national guidelines advocate auditing the cutaneous effects associated with HC [1]. Here, we report a single‐centre experience of 324 HC‐treated MPN patients with long‐term follow‐up, compared to 47 MPN patients who were not prescribed HC. Although our study is limited by retrospective data collection, the outcomes could be reliably identified from the detailed electronic records of patients. The main strength of this study is the large number of patients and the long follow‐up period.

In our cohort, 9.1% of patients developed skin cancers—which equates to a higher incidence than the general Scottish population [10]. This rate is marginally lower than that reported in other studies of MPN patients [7, 8]. This discrepancy may be explained by the fact that other studies reported from populations with greater sun exposure than our north of Scotland population.

By interfering with DNA synthesis, HC has the potential to be mutagenic and therefore provides a plausible biological mechanism for skin cancer development. By acting synergistically with ultraviolet light, HC can increase the risk of development of other non‐cancerous skin changes such as actinic keratoses (AK) [11], with case studies highlighting sustained regression of AKs after discontinuation of HC [12]. However, in our study older age, male gender, type of MPN and previous history of skin cancer were the only statistically significant prognostic risk factors for skin cancer development. HC use was not statistically significant as a prognostic factor for skin cancer development. This is concordant with the findings of Santoro and colleagues [9]. It is however notable that all but one skin cancer occurred in the HC‐treated group, which limits the statistical modelling.

Higher doses and longer exposure to HC were significant to skin cancer risk in one study [13]. Although total dose is an important variable, we were not able to easily assess this in our retrospective study due to frequent adjustments to prescriptions based on blood counts combined with alternative day dosing regimens.

In our practice, we recommend routine skin surveillance and advice of sun protection for all patients prescribed HC. There are no guidelines on the management of the cutaneous side‐effects of HC. The consensus opinion is to discontinue HC after the development of any AK or NMSC [14]. The use of oral retinoids as chemoprevention to mitigate the cutaneous effects of HC use has also been suggested [15]. In our cohort, only 12 patients of 30 with NMSC had their HC discontinued or changed to alternative cytoreductive agents such as anagrelide. The decision to discontinue or change HC treatment in this setting requires consideration of several factors. Managing thrombotic and haemorrhagic complications may be more challenging or burdensome with alternative agents or venesections Arguably; venesection may be preferred in certain PRV patients with higher risk of skin cancers. Furthermore, most BCCs and SCCs can be managed with topical treatments or local excision in minor surgical procedures. There is a low‐associated mortality rate with the development of skin cancers.

In conclusion, we report the incidence of skin cancers in our large cohort of MPN patients. Older age at MPN diagnosis, male gender and type of MPN were statistically significantly associated with the development of skin cancer. Larger multi‐centre studies are required to address whether HC causes skin cancer in this group of patients.

## AUTHOR CONTRIBUTIONS

HGW contributed to the study's conception and design. Data collection and analysis were performed by LL, AM and JE. JE performed statistical analyses. The first draft of the manuscript was written by AM. All authors reviewed and contributed to the writing of the article and approved the final manuscript.

## CONFLICT OF INTEREST

The authors declare they have no conflicts of interest.

## Supporting information



Supporting information.
Figure S1. Survival curve for skin cancer event comparing HC versus non‐HC.
Table S1. Survival curve for skin cancer event comparing HC versus non‐HC.
Figure S2. Survival curve for HC treatment and non‐HC treatment.
Table S2. Survival curve for skin cancer event comparing HC versus non‐HC.
Figure S3. Survival curve for the entire cohort.
Table S3. Survival curve for the entire cohort.

## Data Availability

The data that support the findings of this study are available upon request from the corresponding author. The data are not publicly available due to privacy or ethical restrictions.
