# Supplementary material for: Skin cancer in essential thrombocythaemia and polycythaemia vera patients treated with hydroxycarbamide
Source: EJHaem. 2022 Sep 2;3(4):1305–9. doi: 10.1002/jha2.551 (PMC9713223; doi:10.1002/jha2.551)
Supplement: Supplementary file 1 — Supporting information. Figure S1. Survival curve for skin cancer event comparing HC versus non‐HC. Table S1. Survival curve for skin cancer event comparing HC versus non‐HC. Figure S2. Survival curve for HC treatment and non‐HC treatment. Table S2. Survival curve for skin cancer event comparing HC versus non‐HC. Figure S3. Survival curve for the entire cohort. Table S3. Survival curve for the entire cohort. [file JHA2-3-1305-s001.docx]

**Supplementary data**

**Supplementary Figure 1. Survival curve for skin cancer event comparing HC vs. Non-HC.**

**Supplementary Table 1. Survival curve for skin cancer event comparing HC vs. Non-HC.**

| Group | Hydroxycarbamide | Non-hydroxycarbamide |
| --- | --- | --- |
| Number of rows | 371 | 371 |
| # Censored subjects | 291 | 46 |
| # Events | **33** | **1** |
| Mean until SC in years + 95% CI | **36.3** (31.9-40.6) | **34.6** (31.2-37.9) |

 **Supplementary Figure 2. Survival curve for HC treatment and non-HC treatment.**

**Supplementary Table 2. Survival curve for skin cancer event comparing HC vs. Non-HC.**

| Group | Hydroxycarbamide | Non-hydroxycarbamide |
| --- | --- | --- |
| Number of rows | 371 | 371 |
| # Censored subjects | 294 | 44 |
| # Events | **30** | **3** |
| Mean until SC in years + 95% CI | **37.0** (32.7-41.3) | **39.9** (33.5-46.4) |
|  |  |  |

**Supplementary Figure 3. Survival curve for the entire cohort.**

**Supplementary table 3. Survival curve for the entire cohort.**

| Group | Whole cohort |
| --- | --- |
| Number of rows | 371 |
| # Censored subjects | 338 |
| # deaths/events | 33 |
| Mean survival (years) | **36.7** |
| 95% CI (years) | **33.0-41.5** |
